# A vector representation for phylogenetic trees

**DOI:** 10.1098/rstb.2024.0226

**Published:** 2025-02-20

**Authors:** Cedric Chauve, Caroline Colijn, Louxin Zhang

**Affiliations:** ^1^ Department of Mathematics, Simon Fraser University, Burnaby, British Columbia V5A 1S6, Canada; ^2^ Department of Mathematics, National University of Singapore, Singapore 119076, Singapore

**Keywords:** phylogenetic trees, tree metric, tree rearrangement

## Abstract

Good representations for phylogenetic trees and networks are important for enhancing storage efficiency and scalability for the inference and analysis of evolutionary trees for genes, genomes and species. We propose a new representation for rooted phylogenetic trees that encodes a tree on n ordered taxa as a vector of length 2n in which each taxon appears exactly twice. Using this new tree representation, we introduce a novel tree rearrangement operator, termed an *HOP*, that results in a tree space of linear diameter and quadratic neighbourhood size. We also introduce a novel metric, the *HOP distance*, which is the minimum number of HOPs to transform a tree into another tree. The HOP distance can be computed in near-linear time—a rare instance of tree rearrangement distance that is tractable. Our experiments show that the HOP distance is better correlated to the Subtree-Prune-and-Regraft distance than the widely used Robinson–Foulds distance. We also describe how the proposed tree representation can be further generalized to tree-child networks, showcasing its versatility and potential applications in broader evolutionary analyses.

This article is part of the theme issue ‘"A mathematical theory of evolution": phylogenetic models dating back 100 years’.

## 1. Introduction

Phylogenetic trees and networks are essential mathematical frameworks for modelling the evolution of biological entities [1]. These structures have many applications, facilitating the study of gene, genome and species evolution [2,3], conservation and biodiversity research [4], mapping human migration patterns [5,6] and investigations into the transmission dynamics of infectious diseases such as HIV, COVID-19 and influenza [7,8].

Despite their utility, inferring and processing phylogenetic trees presents significant computational challenges. For inferring phylogenetic trees, both parsimony-based and stochastic models lead to NP-hard problems [9,10], and commonly employed software tools for tackling the tree inference problem, such as RAxML [11], IQ-TREE [12] or BEAST [13], typically navigate the vast tree space by evaluating millions of phylogenetic trees, which can have over hundreds of taxa, to maximize their optimization criteria.

Comparing the topology of phylogenetic trees is another problem that has received significant attention. There are many metrics or distances that have been defined on pairs of phylogenetic trees and that can be used in many contexts, such as comparing trees inferred by different methods or assessing the convergence of Bayesian phylogenetics methods [14]. Many tree metrics are based on the comparison of vectors encoding trees or tree features and are efficiently computable, such as the Robinson–Foulds (RF) distance (the number of clades appearing in one tree but not in the other) [15], the matching distance [16], the Kendall–Colijn distance [17] or the path distance [18]. The matching distance illustrates how a novel representation of trees (through a bijection between phylogenetic trees and perfect matching in graphs) can naturally lead to the definition of a novel tree metric. Other metrics rely on *tree rearrangements*, which are operators that modify phylogenetic trees. A natural distance between two trees is defined as the minimum number of rearrangements needed to transform one into the other. Tree rearrangements are also often used in Bayesian phylogenetics for traversing the tree space. Widely used tree rearrangements include the nearest-neighbour interchange (NNI) [19] and the subtree-prune-regraft (SPR) operators. An SPR removes a subtree from a tree and then re-grafts it on a branch of the remaining tree. An NNI is a special case of an SPR in which the branch onto which the pruned subtree is regrafted and the branch where the subtree was pruned in the original tree are next to each other. Tree rearrangement distances are generally NP-hard to compute (see, for example, [20] for the SPR and NNI distances), except for few exceptions (such as the SPR distance for ranked trees [21]).

While rooted trees are naturally represented by recursive structures, it is common also to consider trees represented as vectors. For instance, the well-known Prüfer code is a bijective encoding of a labelled tree with n vertices using a vector of n−2 integers between 0 and n−1 [22]. A few vector representations have been investigated for phylogenetic trees [16,23–25], leading to novel results. In addition to the example of the matching distance defined in [16], the Phylo2Vec vector presentation of phylogenetic trees [23] was recently investigated for its utility in tree sampling for maximum likelihood tree inference among other applications. Vector representations can also facilitate the application of deep learning methods for tree inference or analysis [24].

Here, we present a vector representation for rooted phylogenetic trees with n ordered taxa, derived from a decomposition technique for trees proposed in [26], although very similar to the representation introduced in [25,27]. In addition, the vector representation is generalized to non-binary phylogenetic trees and the family of tree-child phylogenetic networks [28]. (If there is no inherent ordering to the taxa, alphabetical ordering of the taxa labels can be used, or an ordering can be chosen arbitrarily.) Using our vector representation, we introduce a new tree rearrangement operator and a related distance for tree comparison that, unlike most tree rearrangement-based distances, is computable in near-linear time. Our experiments show that the new distance we introduce better approximates the SPR distance between trees than the widely used RF distance, although we note that these metrics do not rely on taxa ordering.

## 2. Preliminaries

### (a) Phylogenetic trees

**Figure 1.**
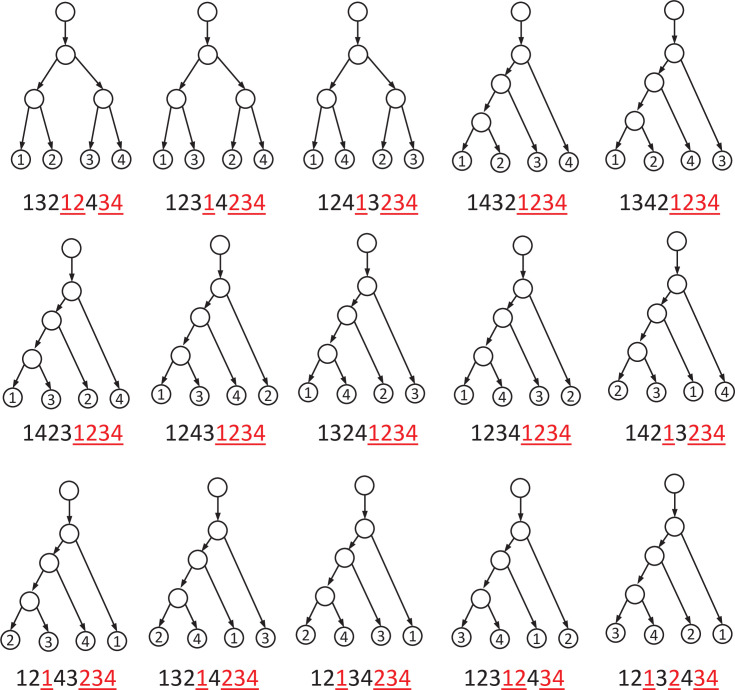
The 15 trees on X={1,2,3,4} and their representations. Here, commas are omitted in the tree representations, in which the second occurrences of the taxa are coloured red and underlined.

Phylogenetic trees are graphic representations of the evolutionary relationships among biological entities (e.g. species, groups or genes). In this paper, we define a *phylogenetic tree* on a set X of n taxa as a rooted directed tree such that (i) the root is of outdegree 1 and indegree 0; (ii) all nodes other than the root are of indegree 1; (iii) the nodes of outdegree 0 are called the leaves, which are in one-to-one correspondence with the n taxa (figure 1); (iv) all non-leaf and non-root nodes have outdegree 2 or more.

Note that, for technical convenience, the definition mentioned above adds an extra node, of outdegree 1, at the root of the tree. The nodes other than the root and the leaves are called *internal nodes*. A phylogenetic tree is *binary* if its internal nodes are of outdegree 2. Consequently, a binary phylogenetic tree on n taxa has one root, n−1 internal nodes and n leaves.

Let T be a phylogenetic tree on taxa X. We use V(T) and E(T) to denote the set of nodes and edges of T, respectively. For any u,v∈V(T), u is the *parent* of v (and v is a *child* of u) if (u,v)∈E(T). The node u is an *ancestor* of v if there is a directed path from u to v. Here, we simply say that ‘u is above v’ and ‘v is below u’ if u is an ancestor of v. We denote by L(T) the set of leaves of T. For u∈V(T), we use c(u) to denote the set of children of u; we use L(u) to denote the set of taxa (i.e. leaves) below u, called the *clade* at the node u. Clades are also called *node clusters*.

In this work, binary phylogenetic trees are simply called trees, in which the leaves are uniquely labelled and internal nodes are not labelled.

### (b) Phylogenetic networks

A *phylogenetic network* on taxa X is a binary rooted acyclic directed graph in which (i) the root is of outdegree 1 and indegree 0; (ii) the nodes of outdegree 0 are called the leaves and are in one-to-one correspondence with the taxa; (iii) all the nodes other than the root and leaves are of indegree 1 and outdegree 2, or indegree 2 and outdegree 1. The nodes with indegree 2 and outdegree 1 are called the *reticulate nodes*. The nodes with indegree 1 and outdegree 2 are called the *tree nodes*.

Phylogenetic networks are discussed only in §3d(ii). Together with this subsection, it has no dependencies with the rest of the paper and may be skipped.

### (c) Vectors and longest common subsequence

Let Σ be an alphabet. A *vector* of length n on Σ is an ordered sequence $\left(v_{1},v_{2},\ldots ,v_{n}\right)$ of elements of Σ. For convenience, we use ‘vector’ and ‘sequence’ interchangeably in this paper. The *length* of a vector s is denoted by |s|. We define by $\left(v_{1},\ldots ,v_{n})+(u_{1},\ldots ,u_{m})=(v_{1},\ldots ,v_{n},u_{1},\ldots ,u_{m}\right)$ the concatenation of vectors.

A vector is a *subsequence* of another if the former can be obtained from the latter by the deletion of zero or more entries. A vector is a *common subsequence* of multiple vectors if it is a subsequence of each vector.

Let S be a set of vectors. A vector is the *longest common subsequence* (LCS) for the vectors S if it is (i) a subsequence of each vector in S and (ii) among all such common subsequences, it has the maximum length. There may be multiple LCSs for the vectors S. We denote by LCS(S) the set of all LCSs of S.

## 3. Results

In this section, we first introduce the novel representation of trees as vectors, together with linear-time algorithms to encode a tree into a vector and decode a vector into a tree. We then introduce the HOP tree rearrangement operator and discuss the properties of the tree space it defines.

### (a) Vector representation of rooted phylogenetic trees

Our representation for phylogenetic trees arises from a path decomposition technique proposed in [26]. By indexing the taxa, we assume that the set of taxa of a given tree is X={1,2,…,n}.

**Definition 3.1**. A vector v on the alphabet X={1,2,…,n} is a tree representation if

(i) for all i∈X, i appears exactly twice in v;

(ii) $v_{1}=1;$

(iii) for all i∈X−{1}, the first occurrence of i in v appears before the second occurrence of i−1;

for all i∈X−{1}, the second occurrence of i in v appears after the second occurrence of i−1.

**Remark**. For clarity, we will use $\underline{i}$ to represent the second occurrence of i in a tree representation.

For example, $\left(1,3,2,\underline{1},\underline{2},4,\underline{3},\underline{4}\right)$ is a tree representation for n=4. However, $\left(1,3,\underline{1},2,4,\underline{2},\underline{3},\underline{4}\right)$ is not, as the first occurrence of 2 appears after the second occurrence of 1. There are 15 trees on four taxa, shown in figure 1 with their respective vector representations.

Let min⁡(u) denote the smallest taxon of the clade L(u) consisting of all descendant taxa of u. We encode a tree into a vector using the following algorithm.


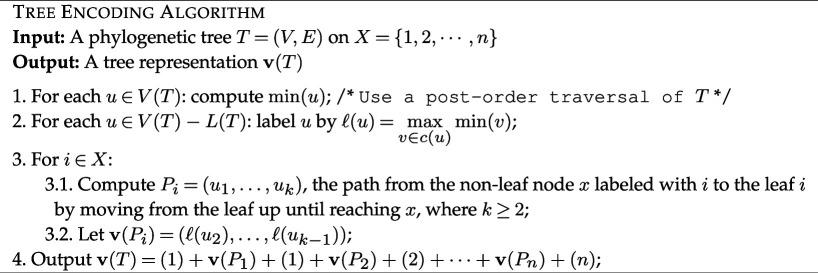


**Remarks**. (i) Following [26], we call the vector $𝐯(P_{i})$ defined in the algorithm the *lineage taxon sequence* (LTS) of taxon i, and we call a tree representation an *LTSv representation*.

(ii) By labelling the internal nodes of a tree T, the tree is decomposed into a set of n disjoint paths. Conversely, if these paths are glued together using the node labelling, the original T is obtained. We further encode the paths into LTSs for taxa and concatenate them to obtain the LTSv representation of T. Therefore, in the LTSv representation 𝐯(T), the first occurrence of each taxon represents an internal node. The second occurrence of each i is used to separate the LTSs $𝐯(P_{i})$ and to represent the leaf i for each 1≤i≤n.

**Lemma 3.1**. *The Tree Encoding Algorithm defines an injective correspondence from the binary phylogenetic trees on* X *(with an ordering of* X*) to LTSv representations on* X*. The algorithm can be implemented in* Θ(n) *time.*

*Proof*. We first prove that after step 2, for a tree T, the n non-leaf nodes of T are uniquely labelled by X. Consider a non-root node x with ℓ(x)=i. Let $x_{1},x_{2}$ be the two children of x, with $\min(x_{1})=i>\min(x_{2})=j$, and let $T_{x_{i}}$ denote the subtree consisting of $x_{i}$ and all the nodes below $x_{i}$ for i=1,2. As the leaf i is not in $T_{x_{2}},$ none of the nodes in $T_{x_{2}}$ is labelled by i. For every non-leaf node y in $T_{x_{1}}$, by definition of min⁡, at least one of its children z satisfies that min⁡(z)>i, so ℓ(z)>i. This implies that none of the nodes in $T_{x}$ are labelled by i except x. Moreover, by construction, the root node is labelled with 1, and any node above x is labelled with the integer j or an integer that is not below x. Since there are n non-leaf nodes in T, every i∈X is the label of a unique non-leaf node in X. Therefore, (i) $P_{i}$ and $𝐯(P_{i})$ are uniquely defined, (ii) $v_{1}=1$ (definition 3.1(ii)), and (iii) every i∈X appears exactly twice in 𝐯(T) (definition 3.1(i)), as each i appears once in a unique $𝐯(P_{j})$ for a unique j<i and is appended after $v(P_{i})$

**Figure 2.**
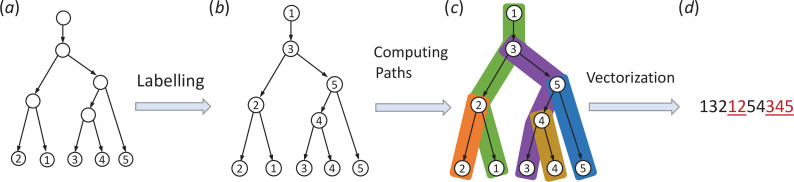
An illustration of the encoding of a phylogenetic tree into an LTSv representation. (*a*) A phylogenetic tree T on X={1,2,3,4,5}. (*b*) The labelling of internal nodes (step 2 of the encoding algorithm). (*c*) The decomposition of the tree into the paths from the non-leaf node labelled i to the leaf i. (*d*) The LTSv representation 𝐯(T), where the second copy of each taxon is underlined and coloured.

. Since T can be restored by gluing the paths $P_{i}$ together using the labels of their first nodes (figure 2), different trees have different LTSv representations.

To show that points (iii) and (iv) of definition 1 are satisfied, consider i∈X−{1}. By step 4, every element of 𝐯(T) either originates from an internal node on an LTS $𝐯(P_{j})$ for some j (in which case it is called an *internal* occurrence) or from a term (i) (in which case it is called a *leaf* occurrence). Let $P_{i}=(u_{1},\ldots ,u_{k})$; by construction, k≥2 and $u_{1}$ and $u_{k}$ are the only vertices labelled with i. By construction, the node $u_{1}$ belongs to a unique other path $P_{j}$ for some j<i∈X and cannot be the first or last node on this path. From the fact j<i, one can deduce that the first occurrence of i is internal and the second occurrence is a leaf occurrence. This implies that the leaf occurrences appear increasingly in 𝐯(T) (definition 1(iv)). Together with j≤i−1, one has that $𝐯(T)=(1)\ldots 𝐯(P_{j})\ldots (i-1)\ldots (i)\ldots$: the first (internal) occurrence of i appears before the second (leaf) occurrence of i−1 (definition 1(iii)).

Last we consider the time complexity. Step 1 (computing min⁡) can be achieved in a single post-order traversal of T. Step 2 requires constant time per node, as each node has at most two children. Computing $P_{i}$ (step 3.1) can be done in time linear in $|P_{i}|$ provided every node knows its parent. This is achieved by starting from leaf i and moving towards the root until reaching the internal node that is labelled with i, which is already on a path $P_{j}$ for some j<i. Step 3.2 requires at most $|P_{i}|$ time, as $𝐯(P_{i})$ consists of the labels of the nodes in $P_{i}$. Since each internal node with i is visited only twice—once for computing the path $P_{j}$ containing the node, and again for computing the path $P_{i}$—the total time taken by step is 2n. Clearly, step 4 requires time Θ(n). This finishes the proof of lemma 1.

For decoding, we compute from an LTSv representation on X={1,2,…,n} the edge set, E(T), of a tree T on X, where an edge is an ordered pair (a,b) where a is the parent of b. To represent the edges, we will use the integer n+i to denote the non-leaf node of T corresponding to the first occurrence of i in the LTSv representation, for each 1≤i≤n. For an LTSv representation $𝐯=(v_{1},\ldots ,v_{2n})$ and $v_{i}=x\in X$, we denote by o(i)=1 (resp. o(i)=2) if $v_{i}$ is the first (resp. second) occurrence of some x in v. For example, in the LTSv representation $\left(1,3,2,\underline{1},\underline{2},4,\underline{3},\underline{4}\right)$, the third character is the first occurrence of 2 and hence o(3)=1. Since the fourth character is the second occurrence of 1,o(4)=2. 
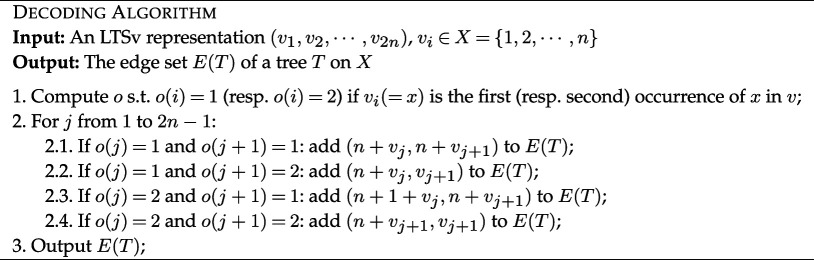


**Example**. Figure 2 provides an example of encoding a tree into an LTSv representation. In the other direction, the table below displays the edges computed using the decoding algorithm applied to the LTSv representation $\left(1,3,2,\underline{1},\underline{2},5,4,\underline{3},\underline{4},\underline{5}\right)$ on X={1,2,3,4,5} (n=5).

**Table:** 

j	$v_{j},v_{j+1}$	$o(v_{j}),o(v_{j}+1)$	edge
1	1,3	1,1	(6,8)
2	3,2	1,1	(8,7)
3	$2,\underline{1}$	1,2	(7,1)
4	$\underline{1},\underline{2}$	2,2	(7,2)
5	$\underline{2},5$	2,1	(8,10)
6	5,4	1,1	(10,9)
7	$4,\underline{3}$	1,2	(9,3)
8	$\underline{3},\underline{4}$	2,2	(9,4)
9	$\underline{4},\underline{5}$	2,2	(10,5)

One can verify that, up to the labels of the internal nodes, the tree defined by this set of edges is the tree shown in figure 2*a*.

**Remark**. An LTSv representation $𝐯=(v_{1},v_{2},\cdots \;,v_{2n})$ is the concatenation of n vectors, each composed of a maximal (possibly empty) prefix of entries x such that o(x)=1 followed by an entry y such that o(y)=2: in the example given above $\left(1,3,2,\underline{1},\underline{2},5,4,\underline{3},\underline{4},\underline{5})=(1,3,2,\underline{1})+(\underline{2})+(5,4,\underline{3})+(\underline{4})+(\underline{5}\right)$. We denote by ${𝐯}_{i}$ the vector ending by $\underline{i}$ minus its last element (in the example given above ${𝐯}_{1}=(1,3,2)$ and ${𝐯}_{3}=(5,4)$); note that by definition, ${𝐯}_{n}$ is empty. We call $\left({𝐯}_{1},\ldots ,{𝐯}_{n-1}\right)$ the *canonical decomposition* of 𝐯. If 𝐯 is the LTSv representation of a tree T resulting from the Tree Encoding Algorithm, by construction, ${𝐯}_{i}$ is the vector $𝐯(P_{i})$.

**Lemma 3.2**. *The Tree Decoding Algorithm defines an injective correspondence from the LTSv representations on* X *to the binary phylogenetic trees on* X*. It can be implemented in* Θ(n) *time.*

*Proof*. Let $\left({𝐯}_{1},\ldots ,{𝐯}_{n-1}\right)$ be the canonical decomposition of $𝐯=(v_{1},v_{2},\cdots \;,v_{2n})$. We let ${𝐯}_{i}^{\prime }={𝐯}_{i}+(i)=(v_{i_{1}},v_{i_{2}},\cdots \;,v_{i_{|{𝐯}_{i}|}},i)$. Clearly, ${𝐯}_{n}^{\prime }=(n)$.

By steps 2.1 and 2.2, every ${𝐯}_{i}^{\prime }$ of length greater than 1 is decoded into a directed path $\left(n+v_{i_{1}},\ldots ,n+v_{i_{|{𝐯}_{i}|}},i\right)$. This results in n paths with a total number of n edges, where, by construction, leaves belong to X and internal nodes are uniquely labelled by {n+1,…,2n}. Step 2.4 creates an edge (n+i,i) for some i∈X whose second occurrence in 𝐯 belongs to a sub-vector of length 1. Step 2.3 applies to a ${𝐯}_{i}^{\prime }$ of length greater than 1. In this case, we create an edge from n+i to $n+v_{i_{1}}$, which connects the internal node labelled with n+i to the first node of the path ending by i∈X. Since the examined symbol is the second occurrence of i−1, i is 1 plus the examined symbol.

This construction of connecting directed path results in an acyclic graph with 2n nodes and 2n−1 edges, which is a tree with 2n nodes uniquely labelled over {1,…,2n}. Node n+1 is the only node with indegree 0 and has outdegree 1, so is the root of the tree. By steps 2.3 and 2.4, every other internal node has outdegree 2. Last, the only nodes of outdegree 0 are the nodes labelled over X. So by removing the labels of the internal nodes, one has a binary phylogenetic tree on X. The construction is uniquely determined by the decomposition into sub-vectors, which is unique to every LTSv representation and so is injective.

Lastly, step 1 takes linear time and linear space if we update the occurrence of each $v_{i}$ from left to right, one at a time. With the vector o, step 1 takes linear time. Thus, the Decoding Algorithm takes linear time.

**Theorem 3.1**. There is a one-to-one correspondence between LTSv representations on X={1,2,…,n} and the binary phylogenetic trees on X.

*Proof*. By lemma 3.1, the vector computed by the encoding algorithm satisfies definition 1. In addition, let 𝐯(T) be the LTSv representation of a tree T. Following the proof of lemma 3.2, we can see that the decoding algorithm computes each $𝐯(P_{i})$ (which consists of edges defined in steps 2.1 and 2.2) from 𝐯(T) and the edges connecting these paths (defined in steps 2.3 and 2.4). Therefore, the decoding algorithm outputs T from 𝐯(T).

**Remarks.**

- In [25], Pons & Batle introduce a vector representation for rooted phylogenetic trees that is very similar to the one introduced above. Indeed, if we denote by $v_{PB}(T)$ this representation, it is straightforward to show that $v_{PB}(T)=v(P_{1})+(2)+v(P_{2})+(3)+\cdots +(n)+v(P_{n})$. This implies that there is a simple bijection between $v_{PB}(T)$ and 𝐯(T), which is defined as $\left(1)+𝐯(P_{1})+(1)+𝐯(P_{2})+(2)+\cdots +𝐯(P_{n})+(n\right)$.
- The tree representation can also be used for unrooted phylogenetic trees by encoding taxa as 0,1,⋯,n and rooting the tree at the leaf 0.
- By theorem 3.1, $𝐯(T_{1})\neq 𝐯(T_{2})$ for two distinct trees $T_{1}$ and $T_{2}$ on X={1,…,n}. If T is a tree with taxa being different from X, the results described above apply if an indexing σ of the taxa is provided, allowing the taxa to be mapped to X. Consequently, different indexing of taxa can result in different vectors for the same tree.
- From a practical point of view, the vector encoding described above can be used to save trees on disk in a slightly more efficient way than the widely used Newick format. We refer to electronic supplementary materials, part (c) for a description of the disc encoding of trees and to §4 for an illustration of this claim.

### (b) The HOP rearrangement operator

**Figure 3.**
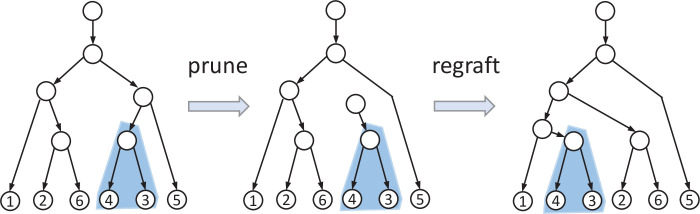
An illustration of the SPR operator.

Widely used tree rearrangement operators used for traversing the space of phylogenetic trees include the NNI and the SPR. The SPR operator is illustrated in figure 3. The NNI rearrangement is a special type of SPR, where the branch of the pruned subtree and the branch where it is regrafted are co-localized in the tree.

The space of phylogenetic trees can be explored by moving from a tree T to a tree that differs from T by a single rearrangement; the set of such trees is the *neighbourhood* of T. The NNI-neighbourhood of a tree T contains Θ(n) trees, while the SPR-neighbourhood contains $\Theta (n^{2})$ trees. The diameter of the tree space defined by the NNI operator is bounded above by Θ(nlog⁡(n)), while the diameter of the space defined by the SPR operator is $n-\Theta (\sqrt{n}))$ [29,30] (see [31] for a survey).

Here, using the LTSv representation of a tree, we introduce a new tree rearrangement operator, which is a special sub-type of SPR, that we call the *HOP operator*. As we will see, the HOP operator defines a tree space of diameter Θ(n), with every tree having a neighbourhood of size $O(n^{2})$.

**Figure 4.**
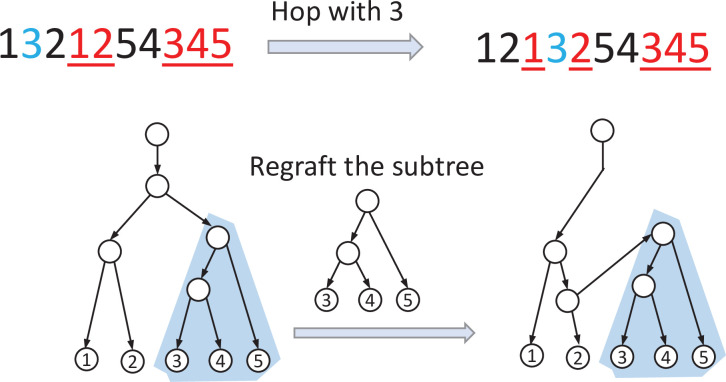
An illustration of the HOP operator and the proof of proposition 3.1. Here, the HOP moves 3 from its original position (before 2) to the new position (before $\underline{2}$) (top). This move’s effect on the tree is equivalent to an SPR rearrangement on the tree represented by the vector (below).

**Definition 3.2**. Let 𝐯 be an LTSv representation on X={1,2,⋯,n}. We use i and $\underline{i}$ to denote, respectively, the first and the second occurrences of i in 𝐯, respectively, for each i∈X. A *HOP rearrangement* on i∈X,i>1 in 𝐯 consists in moving i to a new position located between 1 and $\underline{i-1}$ in 𝐯 (see figure 4).

**Proposition 3.1**. *Let* T *be a phylogenetic tree on taxa* X={1,2,⋯,n}*, with LTSv representation* $𝐯(T)=v_{1}v_{2}\cdots v_{2n}$.

- *Any HOP rearrangement* 𝐯(T) *is an SPR rearrangement on* T*.*
- *The HOP graph, whose vertices are all LTSv representations for trees over* X *and whose edges connect pairs of LTSv representations that differ by a single HOP move, is connected.*
- *The size of the HOP neighbourhood of* 𝐯(T) *in the HOP graph is quadratic in* n*.*

*Proof*. (i) Recall that 𝐯(T) is derived from a decomposition of T into n paths $P_{1},P_{2},\cdots \;,P_{n}$, where $P_{i}$ starts from the internal node u that is represented by the first occurrence of i in 𝐯(T) to the leaf i (figure 2c). Let $u^{\prime }$ be the child of u on the path $P_{i}$. Consider the HOP that moves element i to the position before $v_{j}$. Deleting the first occurrence of i from its current position from 𝐯(T) is equivalent to pruning the subtree $T_{u^{\prime }}$ of T that is rooted at $u^{\prime }$. Re-inserting i before $v_{j}$ is equivalent to regrafting the subtree $T_{u^{\prime }}$ on the edge entering the node x corresponding to $v_{j}$ on the path P containing x. For example, in figure 4, the HOP move is applied to 3. The path $P_{3}$ for the leaf 3 in the path decomposition of the tree T is from the unique child of the root to leaf 3; the path $P_{2}$ consists of leaf 2 and its parent in the tree. The constraint that the new position of the first occurrence of i must not be after $\underline{i-1}$ in 𝐯(T) imposes that this regrafting occurs on a path $P_{k}$ for which k≤i−1; by construction, none of these paths is in the subtree rooted at $u^{\prime }$, so this regrafting is a valid SPR.

(ii) Any LTSv representation can be transformed into the LTSv representation of the caterpillar tree, $\left(1,2,\cdots ,n,\underline{1},\underline{2},\ldots ,,\underline{n}\right)$, using the following sequence of HOP moves: move 2 right after 1, then move 3 right after 2 and so on, until moving n right after n−1. Since the HOP move is a reversible operator, this proves that the graph defined by the HOP operator over the space of tree representations is connected.

(iii) Let $p_{k}$ denote the position in 𝐯(T) of $\underline{k}$. By definition of the LTSv representation, $p_{k}\geq 2k+1$ because—for each j=1,…,k−1—it has two occurrences before $\underline{k}$ and the first occurrence of k and k+1 are also left to $\underline{k}$. Moreover, for a given k, by the definition of the HOP operator, the first occurrence of k can be moved to any position between 1 and $p_{k-1}$ except for its current position using one HOP move. So the number of positions where k∈{2,…,n} can be moved by a HOP is $p_{k-1}-2$. This implies that the number of possible HOP rearrangements is $\sum {\;}_{2\leq k\leq n}(p_{k-1}-2)$, which is at least $\sum_{2\leq k\leq n}[2(k-1)-1]=(n-1{)}^{2}$ and at most $2(n-1{)}^{2}$ because $p_{k}\leq 2n$ for each k. Last, we need to account for overcounting owing to the fact that two different HOPs could result in the same LTSv representation. This can occur only for the following case: moving $v_{t}$ behind $v_{t+1}$ in 𝐯(T) and moving $v_{t+1}$ before $v_{t}$ in 𝐯(T) result in the same vector $v_{1}v_{2}\cdots v_{t-1}v_{t+1}v_{t}v_{t+2}\cdots v_{2n}$, where $v_{t}$ and $v_{t+1}$ are the first occurrences of two leaf labels in 𝐯(T). Therefore, there are at most n such pairs of HOP moves that produce the same LTSv representations. This proves that the size of the HOP neighbourhood of the tree T on n taxa is quadratic in n.

**Remark.** As defined above on phylogenetic trees and LTSv representations on taxa X={1,2,…,n}, exploring the space of LTSv representations and the space of phylogenetic trees using the HOP graph defined in proposition 3.1(ii) is equivalent. However, for phylogenetic trees on an arbitrary set of taxa, the HOP graphs on LTSv representations induced by different taxon orders can be different and there is not a unique HOP graph on trees, but one per fixed taxon order. Note, however, that asymptotic properties such as the diameter of the HOP graph or the upper bound on the neighbourhood size hold independently of the taxon order.

### (c) The HOP distance

Both NNI and SPR define a notion of distance between two trees, which is the minimum number of rearrangements required to transform one tree into the other. Both of these distances are tree metrics and are NP-hard to compute exactly [31]. Similarly to the NNI and SPR, we can define a distance based on the HOP rearrangement. When considering phylogenetic trees on taxa X={1,2,…,n}, this HOP distance defines a metric both on LTSv representations and on phylogenetic trees. However, when considering phylogenetic trees on an arbitrary set of unique taxa, the HOP distance is a metric on LTSv representations for a fixed taxon order, and thus only on phylogenetic trees augmented by a fixed taxon order.

**Definition 3.3**. *Let* 𝐮 *and* 𝐯 *be two LTSv representations on* X={1,2,⋯,n}*. The HOP distance* $d_{\mathrm{HOP}}(𝐮,𝐯)$ *is the minimum number of HOPs necessary to transform* 𝐮 *into* 𝐯*.*

In the rest of this section, we show that the HOP distance is a metric that can be computed in O(nlog⁡n) time, through a variation of the LCS problem.

**Definition 3.4**. *Let* 𝐮 *and* 𝐯 *be two LTSv representations on* X={1,2,⋯,n}, *with* $\left({𝐮}_{1},\ldots ,{𝐮}_{n-1}\right)$ *and* $\left({𝐯}_{1},\ldots ,{𝐯}_{n-1}\right)$ *being their canonical decompositions, respectively. The HOP similarity is defined as*


$${Sim}_{HOP}(u,v)=\sum_{1\leq i\leq n-1}|LCS(u_{i},v_{i})|,$$

where $|\mathrm{LCS}({𝐮}_{i},{𝐯}_{i})|$ is the length of an LCS of ${𝐮}_{i}$ and ${𝐯}_{i}$.

Consider the LTSv representations of the first two trees in figure 1: $u=(1,3,2,\underline{1},\underline{2},4,\underline{3},\underline{4})$ and $v=(1,2,3,\underline{1},4,\underline{2},\underline{3},\underline{4})$. We use ϵ to denote the empty sequence. The canonical decomposition of 𝐮 and 𝐯 are (132,ϵ,4,ϵ) and (123,4,ϵ,ϵ), respectively. Thus, their HOP similarity score is |LCS(132,123)|+|LCS(ϵ,4)|+|LCS(4,ϵ)|+|LCS(ϵ,ϵ)|=2.

**Theorem 3.2**. *Let* 𝐮 *and* 𝐯 *be two LTSv representations on* X={1,…,n}*. Then,*


$$d_{\mathrm{HOP}}(𝐮,𝐯)=n-{\mathrm{Sim}}_{\mathrm{HOP}}(𝐮,𝐯).$$

*Proof*. First, 𝐮=𝐯 if and only if ${\mathrm{Sim}}_{\mathrm{HOP}}(𝐮,𝐯)=n$. Let $k={\mathrm{Sim}}_{\mathrm{HOP}}(𝐮,𝐯)$. An HOP operator moves a single element in an LTSv representation, which implies that it can increase the HOP similarity by at most 1. This implies that $d_{\mathrm{HOP}}(𝐮,𝐯)\geq n-k$.

Next, let the canonical decomposition of 𝐮 and 𝐯 be $\left({𝐮}_{1},{𝐮}_{2},\cdots {𝐮}_{n}\right)$ and $\left({𝐯}_{1},{𝐯}_{2},\cdots {𝐯}_{n}\right)$, where ${𝐮}_{n}={𝐯}_{n}=\epsilon$. For each i≤n−1, we fix an LCS $x_{i1}x_{i2}\cdots x_{it_{i}}$ of ${𝐮}_{i}$ and ${𝐯}_{i}$, where $t_{i}=|\mathrm{LCS}(u_{i},v_{i})|$. An element y of ${𝐮}_{i}$ is said to participate in the dissimilarity of 𝐮 and 𝐯 if $y\neq x_{ij}$ for any j from 1 to t. Similarly, we can define the elements of $v_{i}$ that participate in the dissimilarity of 𝐮 and 𝐯. Let $Y_{i}$ and $Z_{i}$ denote the elements in ${𝐮}_{i}$ and ${𝐯}_{i}$ that participate in the dissimilarity of 𝐮 and 𝐯, respectively. Since each integer i appears exactly once in ${𝐮}_{1}+{𝐮}_{2}+\cdots +{𝐮}_{n}$ and in ${𝐯}_{1}+{𝐯}_{2}+\cdots +{𝐯}_{n}$, $\cup_{1\leq i\leq n}Y_{i}=\cup_{1\leq i\leq n}Z_{i}$, containing exactly n−k integers. For any $z\in \cup_{1\leq i\leq n}Z_{i}$, the integer z appears before $\underline{z-1}$ in both representation 𝐮 and 𝐯. Therefore, we can apply one HOP rearrangement on 𝐯 to move z from its position in 𝐯 to the corresponding position in 𝐮, resulting in an LTSv representation $v^{\prime }$ such that ${\mathrm{SIM}}_{\mathrm{HOP}}(u,v^{\prime })={\mathrm{SIM}}_{\mathrm{HOP}}(u,v)+1$. Using this rule, we can find a sequence of n−k HOP moves that transforms 𝐮 into 𝐯 using the following procedure:

— Let $v_{i}=x$ be the first element in 𝐯 that participates in the dissimilarity. So $\left(u_{1},\ldots ,u_{i-1})=(v_{1},\ldots ,v_{i-1}\right)$ and x is not right next to $u_{i-1}$.
— Moving the first occurrence of x in 𝐮 in the position just after $u_{i-1}$ is a valid HOP rearrangement (as it moves x towards the left) that does increase the HOP similarity by 1, resulting in $\left(u_{1},\ldots ,u_{i})=(v_{1},\ldots ,v_{i}\right)$.
— This can be iterated until both LTSv representations are equal, in exactly n−k HOP rearrangements applied to the n−k elements of 𝐮 that participate in the dissimilarity.

This implies that $d_{\mathrm{HOP}}(𝐮,𝐯)\leq n-k$.

**Remark**. Let $T_{1}$ and $T_{2}$ be the phylogenetic trees on X and let their LTSv representations be 𝐮 and 𝐯, respectively. The vector $LCS(u,v)=(LCS(u_{1},v_{1}),\;\underline{1},\;LCS(u_{2},v_{2}),\;\underline{2},\;\cdots ,\;LCS(u_{n-1},v_{n-1}),\;\underline{n-1},\;\underline{n})$ defines a forest over X whose trees appear as subtrees in both $T_{1}$ and $T_{2}$.

Continuing the example given before theorem 3.2, we have $LCS(u,v)=(1,3,\underline{1},\underline{2},\underline{3},\underline{4})$, which encodes the forest consisting of three subtrees (1,3),(2),(4) in Newick format.

**Proposition 3.2.**

- *The HOP distance between two LTSv representations on* n *taxa can be computed in* O(nlog⁡n) *time.*
- *For a given taxon order, the diameter of the HOP tree space for trees on* n *taxa is* Θ(n)*.*
- *The HOP distance is a metric in the space of (rooted) phylogenetic trees under a fixed taxon order.*

*Proof*. (i) A vector on X is a *partial permutation* if it satisfies the constraint that each element of X occurs at most once in the vector. For each i<n, ${𝐮}_{i}$ and ${𝐯}_{i}$ are partial permutations of X; let $n_{i}=\max(|{𝐮}_{i}|,|{𝐯}_{i}|)$. Applied to partial permutations ${𝐮}_{i}$ and ${𝐯}_{i}$, the LCS algorithm defined in [32] computes $\mathrm{LCS}({𝐮}_{i},{𝐯}_{i})$ in time $O\left(n_{i}\log(n_{i})\right)$. This implies that LCS(𝐮,𝐯) can be computed in time $\sum_{i=1}^{n-1}O\left(n_{i}\log(n_{i})\right)\leq O\left(\max_{1\leq i\leq n-1}\log(n_{i})\sum_{i=1}^{n-1}n_{i}\right)$. As $\sum_{i=1}^{n-1}n_{i}\leq 2n$, LCS(𝐮,𝐯) can be computed in time O(nlog⁡n).

(ii) By definition, the HOP similarity is non-negative. By theorem 3.2, this implies that the HOP distance is at most n. Moreover, consider the LTSv representations (1,2,…,n,1,2,…,n) and (1,2,1,3,2,…,n−2,n−1,n−1,n); the HOP distance between them is n−2, showing that the diameter of the HOP space diameter is at least n−2.

(iii) For any two LTSv representations 𝐮 and 𝐯, by definition of the LCS, ${\mathrm{SIM}}_{\mathrm{HOP}}(𝐮,𝐯)={\mathrm{SIM}}_{\mathrm{HOP}}(𝐯,𝐮)\geq 0$ and thus $d_{\mathrm{HOP}}(𝐮,𝐯)=d_{\mathrm{HOP}}(𝐯,𝐮)$ and the distance is 0 if and only if 𝐮=𝐯.

For any three tree vectors 𝐮,𝐯,𝐰, there is a sequence of $d_{\mathrm{HOP}}(𝐮,𝐯)$ HOP rearrangements that converts 𝐮 into 𝐯, and there is a sequence of $d_{\mathrm{HOP}}(𝐯,𝐰)$ HOP rearrangements that converts 𝐯 to 𝐰. Combining these two sequences of HOP rearrangements, we obtain a sequence of $d_{\mathrm{HOP}}(𝐮,𝐯)+d_{\mathrm{HOP}}(𝐯,𝐰)$ rearrangements that converts 𝐮 to 𝐰. This implies the triangle inequality $d_{\mathrm{HOP}}(𝐮,𝐰)\leq d_{\mathrm{HOP}}(𝐮,𝐯)+d_{\mathrm{HOP}}(𝐯,𝐰)$. This finishes the proof of proposition 2.

**Remarks.**

- A useful consequence of the ability to compute the HOP distance using the vector LCS(𝐮,𝐯) is that it allows one to compute easily a sequence of LTSv representations defined by the shortest sequence of HOP moves that transform 𝐮 into 𝐯, as described in the proof of theorem 3.2.
- An undesired property of the HOP distance is that the LTSv representation of a tree depends on a specific ordering of the taxa. Consider two trees $T_{1},T_{2}$ on the same arbitrary set X of taxa, and two different orderings $\sigma ,\sigma^{\prime }$ of X. Let 𝐮,𝐯 (resp. ${𝐮}^{\prime },{𝐯}^{\prime }$) be the LTSv representations of $T_{1}$ and $T_{2}$, respectively, under σ (resp. $\sigma^{\prime }$). Then, it can occur that ${\mathrm{Sim}}_{\mathrm{HOP}}(𝐮,𝐯)\neq {\mathrm{Sim}}_{\mathrm{HOP}}({𝐮}^{\prime },{𝐯}^{\prime })$, and so $d_{\mathrm{HOP}}(𝐮,𝐯)\neq d_{\mathrm{HOP}}({𝐮}^{\prime },{𝐯}^{\prime })$. It follows that the HOP distance is not a metric on phylogenetic trees on an arbitrary set of taxa but on trees under a fixed taxon ordering, as stated in proposition 3.2.
- This motivates us to introduce the *mHOP* distance, which we conjecture is NP-hard to compute.

**Definition 3.5**. *The mHOP distance* $d_{\mathrm{mHOP}}(T_{1},T_{2})$ *between two trees* $T_{1}$ *and* $T_{2}$ *is equal to the minimum HOP distance between the LTSv representations of* $T_{1}$ *and* $T_{2}$ *over all possible taxon orders.*

### (d) Generalizations

In this subsection, we outline how to obtain a LTSv representation for arbitrary trees and for a subclass of phylogenetic networks.

#### (i) Tree with node names and branch lengths

Given a tree whose nodes (both internal nodes and leaves) are named and with branch lengths associated with edges, the LTSv representation of T can be augmented to encode the full information of the tree as follows: every entry $v_{i}=x$ is replaced by a triplet $v_{i}=[x:y:\ell ]$, where y is the name of the corresponding node and ℓ the length of the branch entering y from its parent (if length information is available), where y is empty for the first triplet. The taxon order used for the encoding can then be deduced from the order of the entries of 𝐯 corresponding to leaves.

**Figure 5.**
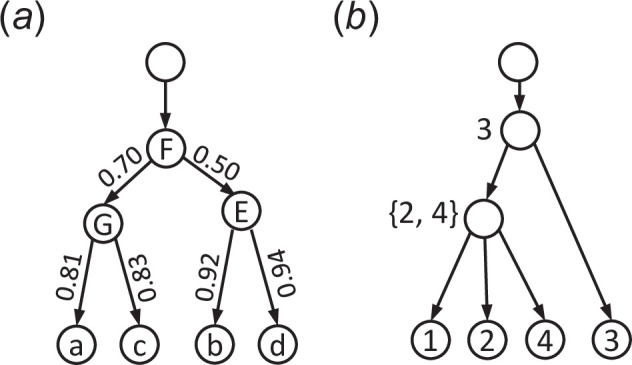
(*a*) A tree with node names and branch lengths. (*b*) A tree with a non-binary node.

For example, if the taxa a,b,c,d are indexed as 1,2,3,4, respectively, the left tree in figure 5 is encoded as:


$$([1::],[2:F:],[3:G:0.70],[\underline{1}:a:0.81],[4:E:0.50],[\underline{2}:b:0.92],[\underline{3}:c:0.83],[\underline{4}:d:0.94]),$$

or simply


$$(1::,2:F:,3:G:0.70,\underline{1}:a:0.81,4:E:0.50,\underline{2}:b:0.92,\underline{3}:c:0.83,\underline{4}:d:0.94).$$

#### (ii) Trees with polytomies

Recall that to obtain the LTSv representation of a binary tree, a node u with children $u^{\prime }$ and $u^{\prime \prime }$ is labelled with the larger of $\min(u^{\prime })$ and $\min(u^{\prime \prime })$.

In a phylogenetic tree, a node of outdegree more than two represents a polytomy where the evolutionary relationships cannot be fully resolved. To obtain a representation of a non-binary tree on taxa X={1,2,⋯,n}, we label each internal node with outdegree two in the same way as for binary trees; we label a node u with children $u_{1},u_{2},\cdots \;,u_{k}$ (k>2) with the subset $\left\{\min(u_{1}),\min(u_{2}),\cdots ,\min(u_{k})\}\setminus \{\min_{1\leq i\leq k}\min(u_{i})\right\}$, which is the subset consisting of all $\min\;u_{i}$ except the smallest one. In this way, the representation of a non-binary tree can be encoded as a sequence of taxa, commas and braces, in which a pair of braces corresponds to a polytomy node in the tree. For instance, $(1,3,\{24\},\underline{1},\underline{2},\underline{3},\underline{4})$ is the representation of the tree with one polytomy node given in figure 5*b*.

For the representation of a non-binary tree on X={1,2,⋯,n}, the four conditions in definition 3.1 also hold. In addition, there is a pair of curly brackets for each non-binary node. Thus, the length of the representation for a tree on X is 2n+2t if there are t non-binary nodes in the tree.

Remark (ii) before lemma 3.1 about the representation for binary trees also applies to the non-binary trees. Therefore, there is one-to-one correspondence between the non-binary trees on X={1,2,⋯,n} and the vector representations.

#### (iii) Tree-child networks

A phylogenetic network is a *tree-child network* if every non-leaf node has at least one child that is not a reticulation, where a reticulation is a node with indegree two and outdegree one. The LTSv representation can be generalized to the family of tree-child networks as follows.

**Figure 6.**
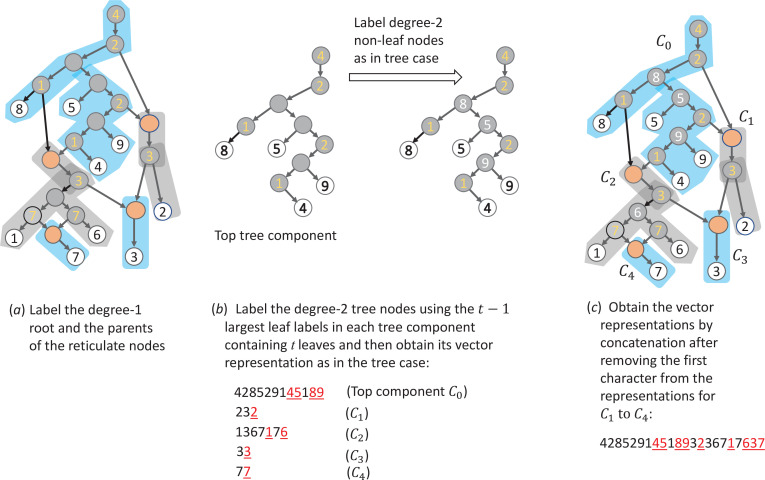
Illustration of the procedure of encoding tree-child networks. The five tree components of the network are highlighted in blue and grey.

A connected component of the forest resulting from removing all the edges entering reticulated nodes from a tree-child network is called a *tree component* of the network. For instance, there are five tree components (shaded) in the network in figure 6. The root of a tree component is either the network root or a reticulation node. Consequently, a tree-child network with r reticulation nodes has r+1 tree components. The tree component rooted at the network root is called the *top component*.

For a tree-child network N on X={1,2,⋯,n}, we obtain the representation for each of its tree components in the same way as in the case of a binary tree. Then, we combine these representations to obtain the representation of N. The order in which the tree components are combined will be specified in the algorithm. This encoding algorithm is summarized as follows:

— Label the degree-1 root by the smallest taxa in the top component. Similarly, label the two parents of each reticulation node y with the smallest taxa in the tree component rooted at y (figure 6*a*).
— If a tree component of the network contains t taxa, it contains t−1 tree nodes whose two children are also tree nodes in the original network (figure 6*b*). We label these tree nodes using one of the t−1 largest taxa in the component and obtain the vector representation of the component using the same rule as in the binary tree case (figure 6*b*).
— We order the components using the vector representations of the tree components inductively. Note that the representation of each component induces an ordering on all the nodes in the component. First, the top component receives index 0. Assume the k components have been indexed as $C_{0},C_{1},\cdots \;,C_{k-1}$. Now, consider all non-indexed components whose root (which is a reticulation node) has its two parents already in the indexed tree components. If both parents are in the same component, say K, then one appears after the other one in the representation of K; the latter is called the second parent. If the parents are in different components, then the parent in the component with the higher index is called the second parent. Now order all the above chosen non-indexed components according to where the second parents are located. If the second parent of the tree component *A* appears in $C_{i}$ and the second parent of the tree component B appears in $C_{j}$ and i<j, A has an index smaller than B. If the second parent of A and B appears in the same tree component $C_{i}$, A has a smaller index than B if the second parent of A appears earlier than that of B in the vector representation of $C_{i}$. Iterate this process until all path components are indexed, which will eventually happen because our networks are connected and acyclic. This step is illustrated in figure 6*c*.
— Assume the r+1 components of the network are indexed as $C_{0},C_{1},\cdots \;,C_{r}$ and the vector representation of $C_{i}$ to be ${𝐯}_{i}$. The vector representation of the network is the concatenation of ${𝐯}_{0}^{\prime },{𝐯}_{1}^{\prime },\cdots \;,{𝐯}_{r}^{\prime }$, where ${𝐯}_{i}^{\prime }$ is obtained by removing the first element of ${𝐯}_{i}$ (figure 6*c*).

The algorithm for decoding an LTSv representation for tree-child networks is similar to that used for trees. It starts with scanning the input LTSv representation 𝐯 from left to right to identify which taxa occur twice and which occur three times. The taxa appearing three times correspond to the reticulation nodes in the output network.

Assume 𝐯 contains n taxa. These taxa are ordered according to their last occurrence from left to right as $x_{1},x_{2},\cdots \;,x_{n}$. We then split 𝐯 into disjoint substrings $v_{1},v_{2},\cdots \;,v_{n}$, which may include the empty strings, such that


$$v=v_{1}+\underline{x}{\;}_{1}+v_{2}+\underline{x}{\;}_{2}+\cdots +v_{n}+\underline{x}{\;}_{n},$$

where $\underline{x}{\;}_{i}$ is the last occurrence of $x_{i}$. Next, for each i, we assume $v_{i}=a_{i1}a_{i2}\cdots a_{ik}$. From each $v_{i}$, construct a path $P_{i}$ with k+2 nodes:


$$z_{i}\to y_{i1}\to y_{i2}\to \cdots \to y_{ik}\to \ell_{i}.$$

Here, $\ell_{i}$ is the leaf representing the taxon $x_{i}$, and $z_{i}$ represents a reticulation node if $x_{i}$ appears three times in 𝐯. We then connect the nodes $y_{ij}$ in $P_{i}$ to the first node $z_{k}$ of the path $P_{k}$ if $a_{ij}$ matches $x_{k}$. If $x_{k}$ occurs twice in 𝐯, contract the node $z_{k}$ for each k. This completes the construction of the output network.

## 4. Experiments

The encoding and decoding algorithm, as well as the HOP similarity algorithm, were implemented in Python and are freely available at https://github.com/cchauve/CEDAR.

### (a) Encoding phylogenetic trees

We generated 1000 random rooted phylogenetic trees, on 100 taxa labelled t1, …, t100, with random branch lengths. The trees were generated with an in-house script starting from a tree with a single edge with a leaf labelled t1 and adding the remaining leaves one by one by uniformly selecting a random edge for attachment; branch lengths were generated randomly between 0 and 1 using the random generator in Python. The encoding of the trees using the LTSv representation as described in §3a requires slightly less disk space (92%) than the Newick encoding.

### (b) HOP neighbourhood size

For each of the 1000 random trees, we computed the size of the HOP neighbourhood (the trees differing from the starting tree by exactly one HOP) based on a vector encoding obtained with taxa ordered according to a post-order traversal of the first tree. The mean HOP neighbourhood size is 12 424 , with a 183 s.d.—the smallest (resp. largest) HOP neighbourhood containing 9958 (resp. 13 000 ) trees. This illustrates that despite the HOP rearrangement operator being a special type of SPR, it defines a neighbourhood of quadratic size.

### (c) HOP distance, RF distance and SPR

In the last experiment, we selected the first 50 random trees from the previous experiments and generated from each selected tree a set of 20 trees obtained by applying 5,10,15,…,100 random SPR rearrangements, respectively. We call the initial trees the *focal trees* and the trees obtained by SPR rearrangements the *SPR trees*.

For every pair $\left(T,T^{\prime }\right)$ where T is a focal tree and $T^{\prime }$ an SPR tree obtained from T, we computed:

— the widely used RF distance between T and $T^{\prime }$, normalized by dividing it by its maximum value (200);
— the HOP distances, normalized by dividing it by its maximum value (100).

To assess the impact of the taxon indexing required us to generate the vector representations of trees on the HOP distance between the trees, we generated 10 random taxa orders and repeated the HOP distance computation for each order.

Electronic supplementary material, figure S1 and table 1 show that the HOP distance correlates better overall with the actual number of SPRs used to generate SPR trees than with the RF distance, especially for SPR trees obtained through a moderate number of SPR rearrangements. However, we observe a larger variation in the HOP distance value when considering 10 random taxa orders, compared to the RF distance, illustrating the effect of the chosen taxa order.

**Table 1.** Pearson correlation between the number of SPR steps and the RF and HOP distances. Every column corresponds to all comparisons between the starting tree and the trees obtained with up to k SPR moves. RF: Robinson–Foulds distance; HOP: HOP distance accumulated over the 10 random taxa orders; mean HOP: mean of the HOP distance over the 10 random taxa orders.

*k*	10	20	30	40	50	60	70	80	90	100
RF	0.815	0.902	0.909	0.913	0.909	0.905	0.900	0.892	0.883	0.874
HOP	0.707	0.867	0.909	0.924	0.928	0.928	0.926	0.921	0.916	0.910
mean HOP	0.840	0.935	0.953	0.956	0.954	0.950	0.944	0.938	0.931	0.923

Since the HOP distance depends implicitly on the taxa order used to generate vector encoding, we examined the variance of HOP distance under the 10 random orders used in our experiments. Electronic supplementary material, figure S2 shows the mean and s.d. of the normalized HOP distance over the 10 random taxa orders. We can observe that taking the mean HOP distance over 10 random taxa orders results in a variation over the 50 trees comparable to what we observe with the RF distance. Table 1 shows that the mean HOP distance correlates much better with the actual number of SPR steps than the RF distance. Given that the HOP distance is very fast to compute, this suggests that applying it with a small number of random taxa orders allows the counterbalancing of the impact of a chosen taxa order on the distance value, at a minimal computational cost.

### (d) HOP distance reveals useful information on tree similarity

In electronic supplementary material, figure S3, two phylogenetic trees depicting 91 fungus species are presented. These trees were constructed by rooting on the branch leading to *Aspergillus aculeatus*, derived from two unrooted trees generated by applying IQ-TREE [12] with different parameters to the same set of sequences taken from the NCBI database, given in [33]. The HOP distance between these two trees is 10, with the species ordered as they appear in the Newick representation of the first tree. The LCS defined by the HOP distance defined a forest of three distinct large subtrees (electronic supplementary material, figure S4);see the remark before proposition 3.2, illustrating a case where a moderate HOP distance translates into large conserved structure.

## 5. Discussion and conclusion

We have introduced a novel representation of phylogenetic trees (with polytomies allowed, and with an ordering of the taxa) as vectors. This representation is slightly more efficient than the Newick format to store trees. In this representation, the internal nodes of a tree are represented by taxa and listed in a topological sorting ordering. This ordering ensures that the orientation of tree branches aligns with the sequence of internal nodes in the representation. Accordingly, as detailed in the electronic supplementary material, parts (a) and (b), this representation enables us to develop an alternative method for counting phylogenetic trees and an algorithm for verifying tree identities through direct comparison of their vector representations. It also leads to an efficient algorithm for computing the RF distance (electronic supplementary material, part (d))). Furthermore, since this vector representation can be extended to tree-child networks, exploring its applications in the network domain presents an intriguing avenue for future research.

We also introduce the HOP rearrangement operator and the HOP distance metric. The HOP rearrangement is implemented by moving a single entry in the vector representation of a tree and is thus easy to implement. Although the HOP operator is a special type of SPR, the neighbourhood size for it remains quadratic in order and the maximum HOP distance between two trees is less than n HOP rearrangements. Unlike most tree rearrangement operators, such as NNI and SPR, the HOP distance can be computed efficiently, in near-linear time. Moreover, given two trees, it is easy to compute the *shortest* sequence of HOP rearrangements that transform a tree into the other. Our experiments suggest that the HOP distance captures the dissimilarity between trees better than the RF distance. Most importantly, the computation of the transformation between two trees allows us to identify the common subtrees shared by the trees, even with thousands of taxa.

One feature of the HOP distance is its dependence on the taxon order used to encode trees into vectors. This characteristic is inherent to many dimensionality reduction techniques in data science, where the focus is on finding a good solution rather than the best one to overcome the challenge of large datasets. For efficient storage, the representation under any taxon order serves the purpose well. For the measurement of the dissimilarity between trees, our experiments show that the variation of HOP distance from different taxa orders is significant, particularly for very similar trees. Consequently, the HOP distance can serve as a viable proxy for the SPR distance, especially for large trees where computing the SPR distance becomes impractical. In cases where the SPR distance approaches the number of taxa, the HOP distance is expected to provide a reliable measure of dissimilarity. Furthermore, for similar trees, the use of the mHOP distance, which minimizes the HOP distance over all possible taxon orders, is recommended. While the NP-hardness of computing the mHOP distance remains unknown, it is plausible to devise a fast heuristic for it by sampling taxon orderings, as demonstrated in [26], for inferring phylogenetic networks.

Lastly, owing to the efficiency of our vector representation in storage and the ease of implementing the HOP operator, there is potential for accelerating the development of machine learning methods for inferring phylogenetic trees. Exploring this research direction holds significant promise.

## Data Availability

Supplementary material is available online at [34].

## References

[1] Felsenstein J. 2004 Inferring phylogenies. Sunderland, MA: Sinauer Associates.

[2] Dayhoff MO, McLaughlin PJ, Barker WC, Hunt LT. 1975 Evolution of sequences within protein superfamilies. Die Naturwissenschaften **62**, 154–161. (10.1007/bf00608697)

[3] Murphy WJ *et al*. 2005 Dynamics of Mammalian Chromosome Evolution Inferred from Multispecies Comparative Maps. Science **309**, 613–617. (10.1126/science.1111387) 16040707

[4] Redding DW, Hartmann K, Mimoto A, Bokal D, Devos M, Mooers AØ. 2008 Evolutionarily distinctive species often capture more phylogenetic diversity than expected. J. Theor. Biol. **251**, 606–615. (10.1016/j.jtbi.2007.12.006) 18313078

[5] Cann RL, Stoneking M, Wilson AC. 1987 Mitochondrial DNA and human evolution. Nature **325**, 31–36. (10.1038/325031a0) 3025745

[6] Ingman M, Kaessmann H, Pääbo S, Gyllensten U. 2000 Mitochondrial genome variation and the origin of modern humans. Nature **408**, 708–713. (10.1038/35047064) 11130070

[7] Boni MF, Lemey P, Jiang X, Lam TTY, Perry BW, Castoe TA, Rambaut A, Robertson DL. 2020 Evolutionary origins of the SARS-CoV-2 sarbecovirus lineage responsible for the COVID-19 pandemic. Nat. Microbiol. **5**, 1408–1417. (10.1038/s41564-020-0771-4) 32724171

[8] Sharp PM, Hahn BH. 2010 The evolution of HIV-1 and the origin of AIDS. Phil. Trans. R. Soc. B **365**, 2487–2494. (10.1098/rstb.2010.0031) 20643738 PMC2935100

[9] Foulds LR, Graham RL. 1982 The steiner problem in phylogeny is NP-complete. Adv. Appl. Math. **3**, 43–49. (10.1016/s0196-8858(82)80004-3)

[10] Roch S. 2006 A Short Proof that Phylogenetic Tree Reconstruction by Maximum Likelihood Is Hard. IEEE/ACM Trans. Comput. Biol. Bioinform. **3**, 92–94. (10.1109/TCBB.2006.4) 17048396

[11] Stamatakis A, Ludwig T, Meier H. 2005 RAxML-III: a fast program for maximum likelihood-based inference of large phylogenetic trees. Bioinformatics **21**, 456–463. (10.1093/bioinformatics/bti191) 15608047

[12] Minh BQ, Schmidt HA, Chernomor O, Schrempf D, Woodhams MD, von Haeseler A, Lanfear R. 2020 IQ-TREE 2: New Models and Efficient Methods for Phylogenetic Inference in the Genomic Era. Mol. Biol. Evol. **37**, 1530–1534. (10.1093/molbev/msaa015) 32011700 PMC7182206

[13] Suchard MA, Lemey P, Baele G, Ayres DL, Drummond AJ, Rambaut A. 2018 Bayesian phylogenetic and phylodynamic data integration using BEAST 1.10. Virus Evol. **4**, y016. (10.1093/ve/vey016) PMC6007674 29942656

[14] Lanfear R, Hua X, Warren DL. 2016 Estimating the Effective Sample Size of Tree Topologies from Bayesian Phylogenetic Analyses. Genome Biol. Evol. **8**, 2319–2332. (10.1093/gbe/evw171) 27435794 PMC5010905

[15] Robinson DF, Foulds LR. 1981 Comparison of phylogenetic trees. Math. Biosci. **53**, 131–147. (10.1016/0025-5564(81)90043-2)

[16] Diaconis PW, Holmes SP. 1998 Matchings and phylogenetic trees. Proc. Natl Acad. Sci. USA **95**, 14600–14602. (10.1073/pnas.95.25.14600) 9843935 PMC24495

[17] Kendall M, Colijn C. 2016 Mapping Phylogenetic Trees to Reveal Distinct Patterns of Evolution. Mol. Biol. Evol. **33**, 2735–2743. (10.1093/molbev/msw124) 27343287 PMC5026250

[18] Steel MA, Penny D. 1993 Distributions of Tree Comparison Metrics—Some New Results. Syst. Biol. **42**, 126–141. (10.1093/sysbio/42.2.126)

[19] Moore GW, Goodman M, Barnabas J. 1973 An iterative approach from the standpoint of the additive hypothesis to the dendrogram problem posed by molecular data sets. J. Theor. Biol. **38**, 423–457. (10.1016/0022-5193(73)90251-8) 4632522

[20] DasGupta B, He X, Jiang T, Li M, Tromp J, Zhang L. 1997 On distances between phylogenetic trees. In ACM-SIAM Symposium on Discrete Algorithms, January 5--7, the Association for Computing Machinery, Inc., New York, USA, pp. 427–436. New York, NY: Association for Computing Machinery, Inc.

[21] Collienne L, Gavryushkin A. 2021 Computing nearest neighbour interchange distances between ranked phylogenetic trees. J. Math. Biol. **82**, 8. (10.1007/s00285-021-01567-5) 33492606 PMC7835203

[22] Prufer H. 1918 Neuer beweis eines satzes uber per mutationen. Archiv der Mathematik und Physik **27**, 742.

[23] Penn MJ, Scheidwasser N, Khurana MP, Duchêne DA, Donnelly CA, Bhatt S. 2024 Phylo2Vec: a vector representation for binary trees. Syst. Biol syae030. (10.1093/sysbio/syae030) 38935520 PMC11958935

[24] Voznica J, Zhukova A, Boskova V, Saulnier E, Lemoine F, Moslonka-Lefebvre M, Gascuel O. 2022 Deep learning from phylogenies to uncover the epidemiological dynamics of outbreaks. Nat. Commun. **13**, 3896. (10.1038/s41467-022-31511-0) 35794110 PMC9258765

[25] Pons M, Batle J. 2021 Combinatorial characterization of a certain class of words and a conjectured connection with general subclasses of phylogenetic tree-child networks. Sci. Rep. **11**, 21875. (10.1038/s41598-021-01166-w) 34750409 PMC8575882

[26] Zhang L, Abhari N, Colijn C, Wu Y. 2023 A fast and scalable method for inferring phylogenetic networks from trees by aligning lineage taxon strings. Genome Res. **33**, 1053–1060. (10.1101/gr.277669.123) 37217252 PMC10538497

[27] Fuchs M, Yu GR, Zhang L. 2021 On the asymptotic growth of the number of tree-child networks. Eur. J. Comb. **93**, 103278. (10.1016/j.ejc.2020.103278)

[28] Cardona G, Rossello F, Valiente G. 2008 Comparison of Tree-Child Phylogenetic Networks. IEEE/ACM Trans. Comput. Biol. Bioinform. **6**, 552–569. (10.1109/TCBB.2007.70270) 19875855

[29] Allen BL, Steel M. 2001 Subtree Transfer Operations and Their Induced Metrics on Evolutionary Trees. Ann. Comb. **5**, 1–15. (10.1007/s00026-001-8006-8)

[30] Li M, Tromp J, Zhang L. 1996 On the Nearest Neighbour Interchange Distance Between Evolutionary Trees. J. Theor. Biol. **182**, 463–467. (10.1006/jtbi.1996.0188) 8944893

[31] St. John K. 2017 Review Paper: The Shape of Phylogenetic Treespace. Syst. Biol. **66**, e83–e94. (10.1093/sysbio/syw025) 28173538 PMC5837343

[32] Hunt JW, Szymanski TG. 1977 A fast algorithm for computing longest common subsequences. Commun. ACM **20**, 350–353. (10.1145/359581.359603)

[33] Shen XX, Li Y, Hittinger CT, Chen XX, Rokas A. 2020 An investigation of irreproducibility in maximum likelihood phylogenetic inference. Nat. Commun. **11**, 6096. (10.1038/s41467-020-20005-6) 33257660 PMC7705714

[34] Chauve C, Colijn C, Zhang L. 2024 Supplementary material from: A Vector Representation for Phylogenetic Trees. Figshare. (10.6084/m9.figshare.c.7599495)39976399

